# Variability in commercial demand for tree saplings affects the probability of introducing exotic forest diseases

**DOI:** 10.1111/1365-2664.13242

**Published:** 2018-08-14

**Authors:** Vasthi Alonso Chavez, Christopher A. Gilligan, Frank van den Bosch

**Affiliations:** ^1^ Department of Biointeractions and Crop Protection Rothamsted Research Harpenden UK; ^2^ Department of Plant Science University of Cambridge Cambridge UK

**Keywords:** demand, forest disease, gross margin, import, model, nursery, tree, variability

## Abstract

1. Several devastating forest pathogens are suspected or known to have entered the UK through imported planting material. The nursery industry is a key business of the tree trade network. Variability in demand for trees makes it difficult for nursery owners to predict how many trees to produce in their nursery. When in any given year, the demand for trees is larger than the production, nursery owners buy trees from foreign sources to match market demand. These imports may introduce exotic diseases.
2. We have developed a model of the dynamics of plant production linked to an economic model. We have used this to quantify the effect of demand variability on the risk of introducing an exotic disease.
3. We find that: (a) When the cost of producing a tree in a UK nursery is considerably smaller than the cost of importing a tree (in the example presented, less than half the importing cost), the risk of introducing an exotic disease is hardly affected by an increase in demand variability. (b) When the cost of producing a tree in the nursery is smaller than, but not very different from the cost of importing a tree, the risk of importing exotic diseases increases with increasing demand variability.
4. *Synthesis and applications*. Our model and results demonstrate how a balanced management of demand variability and costs can reduce the risk of importing an exotic forest disease according to the management strategy adopted. For example, a management strategy that can reduce the demand variability, the ratio of production to import cost or both, optimizes the nursery gross margin when mainly own‐produced trees are commercialized. This can also translate into a reduction of the risk of introducing exotic forest diseases due to the small number of imported trees for sale.

## Abstract 2

Our model and results demonstrate how a balanced management of demand variability and costs can reduce the risk of importing an exotic forest disease according to the management strategy adopted. For example, a management strategy that can reduce the demand variability, the ratio of production to import cost or both, optimizes the nursery gross margin when mainly own‐produced trees are commercialized. This can also translate into a reduction of the risk of introducing exotic forest diseases due to the small number of imported trees for sale.

## Abstract (fr)

1. Se conoce o sospecha que algunos patógenos forestales encontrados en el Reino Unido han sido introducidos a través de material de siembra importado. La industria de viveros es un negocio clave dentro de la red de comercialización forestal. Sin embargo, la demanda comercial de árboles varía frecuentemente. Esto resulta problemático para los viveros quienes deben calcular cuántos árboles necesitan plantar para su comercialización. Cuando la demanda al punto de venta es mayor que la producción del vivero, la demanda es satisfecha con importaciones de fuentes extranjeras. Estas importaciones pueden introducir plagas y enfermedades forestales exóticas.
2. Desarrollamos un modelo de la dinámica de producción forestal vinculado a un modelo económico del vivero para cuantificar el efecto de la variabilidad en la demanda comercial sobre el riesgo de introducir una enfermedad exótica.
3. Nuestro modelo muestra lo siguiente: i. Cuando el costo de producir un árbol en un vivero del Reino Unido es considerablemente menor que el costo de importar un árbol (en el ejemplo presentado es más de dos veces menor al costo de importación), el riesgo de introducir un patógeno forestal exótico apenas se ve afectado por incrementos en la variabilidad de la demanda. ii. Cuando el costo de producción de un árbol es menor, pero no muy diferente del costo de importación, el riesgo de introducir un patógeno forestal exótico incrementa a medida que la variabilidad en la demanda aumenta.
4. *Síntesis y aplicaciones*. Nuestro modelo y resultados demuestran que un manejo equilibrado de los costos y la variabilidad en la demanda comercial de árboles en viveros puede reducir el riesgo de importación de enfermedades forestales exóticas de acuerdo con la estrategia de manejo adoptada. Por ejemplo, una estrategia de manejo que reduzca la variabilidad en la demanda, la relación entre producción y costo de importación, o ambas cantidades, optimiza el margen bruto del vivero cuando se comercializan principalmente árboles de producción propia. Esto también se puede traducir en una reducción del riesgo de introducción de enfermedades forestales exóticas debido a la baja comercialización de árboles importados.

## 1 INTRODUCTION

### 1.1 Invasive forest pathogens and imports

A known pathway for the introduction of invasive forest pathogens is imports of infected seeds, seedlings, and live plants (Klapwijk et al., 2016). Nursery trade involves large‐scale movements of material for the forestry industry generating considerable economic value. However, it is also considered as a pathway for the introduction of tree pests and diseases (EPPO, 2012; Liebhold, Brockerhoff, Garrett, Parke, & Britton, 2012).

Smith et al. (2018) estimate that a total of 267 introduced plant pathogens became established in Great Britain from 1970 to 2013. From these, 10 species affect the forestry sector. About two‐thirds of the total introduced species are native to continental Europe with approximately 50% of the total coming from the Netherlands and 14% from France (Jones & Baker, 2007). North America and Asia follow with about 20% of establishments, 10% from each region (Smith et al., 2018).

Introduced pathogens from across continents can have catastrophic effects. For example, Dutch elm disease (*Ophiostoma novo‐ulmi*) native to Eastern Asia was introduced to the UK via imported, infected elm logs from Canada. This disease killed over 45 million trees in Britain, leaving very few elm trees alive (Brasier, 2008). However, even pathogens introduced from nearer regions can be devastating. One example is the oak processionary moth (*Thaumetopoea processionea*). This pest was likely introduced to England through the movement of live oak plants in trade from continental Europe (Forestry Commission GB, 2016). The oak processionary moth is native of southern Europe and affects the health of oak trees, people, and animals. Although this pest is confined to a relatively small area, it is subject to a government‐led program of survey and management due to its’ potential health consequences.

### 1.2 Ecological importance of forests

Ecologically speaking, forests play a very important role. For example, they help mediate against global climate change, improve air quality, and reduce the damage from floods. Woodlands also harbour a large number of species, providing an important biodiversity resource for future generations (Woodland Trust & Europe Economics, 2017). However, due to woodland fragmentation and unreliable natural regeneration, woodland regeneration has mainly been conducted by the planting of nursery‐raised tree seedlings (Whittet, Cottrell, Cavers, Pecurul, & Ennos, 2016).

### 1.3 Size and extent of industry

The UK currently imports around 80% of its wood and wood products. It was the second largest net importer of forest products in 2015 with net imports of US$9 billion, behind China (Forestry Commission, 2018). Despite this, in 2010, the forestry and primary timber processing sector contributed £1.7 billion in gross value added to the UK economy, supporting around 43,000 jobs and employed around 14,000 people in more than 3,000 enterprises (DEFRA, 2013), and in 201l the UK produced £635.8 m of forestry goods (Woodland Trust, & Europe Economics, 2017).

In the UK, the nursery industry is formed of several types of businesses such as wholesale traders, garden centres, retail nurseries, landscape businesses and nursery growers, among others. Here, we focus on forest growers. Forest growers belong to a group of production nurseries that use seed or unfinished stock to grow plants until marketable at a more mature stage. Growers often trade plant material in addition to selling own product (CONFOR, 2013).

Since the early 1900s, the UK woodland area has increased from 5% to 13% total land area (Forestry Commission GB, 2017). From 1919 to 1987, most of the forest expansion was dominated by conifer planting to support an increase in domestic timber demand (Woodland Trust, 2011). In 1988, a new forestry grant scheme (The Woodland Grant Scheme) was introduced, paying nearly twice as much for broadleaf woodland as conifers. Therefore, broadleaves have received much more attention since then (Forestry Commission, 2018).

### 1.4 Factors affecting variability

One of the main challenges for nursery businesses is uncertainties in the market demand (Downing, 2012; Whittet et al., 2016).

Most tree planting schemes in the UK are eligible for subsidy support through the Common Agricultural Policy of the European Union. These schemes often require locally sourced seed of specific species and provenance. These seeds, plants, and cuttings can be sourced and grown in nurseries in the UK and other European nurseries. If EU regulations are followed, nurseries can trade amongst themselves and European nurseries to meet demand (Forestry Commission, 2007; Whittet et al., 2016).

Growers often rely on grants, schemes, and private sector orders to maintain their businesses. These grants and schemes can change in short periods of time, depending on the market needs, government changes, grant schemes reforms, and stakeholders’ priorities among others (Downing, 2012; House of Commons, 2016). Therefore, forest managers often give short notice of their plant requirements to nursery owners. This is even though nurseries require up to 3 years to produce a tree seedling depending on the species, and longer if targeted seed collection is required (Whittet et al., 2016).

Forest growers have limited planting land. As finished stock can take up to 3 years to grow, they must determine several years in advance which species to grow. If grant schemes and demand do not change over several years, nursery stock can be matched to demand and nurseries can provide their own finished stock. If market demand changes more abruptly, for example in intervals of 1 or 2 years, growers must rely on trade with other local and foreign sources. Moreover, sharp changes in demand may result in the destruction of large quantities of unwanted trees to make space for new planting. A good review of how changes in seedling demand affect forest growers can be found in Whittet et al. (2016). As variability in the market demand hampers growers’ ability to predict the number of trees required to satisfy the demand in any given year, nurseries often protect themselves from monetary losses by restricting the number of trees per species they produce, aiming to reduce the number of unsold trees. When in any given year the demand is larger than the production, nursery owners buy trees from foreign sources to match market demand. These imports may introduce exotic diseases. An important question thus is the extent to which this demand variability contributes to the risk of introducing new tree diseases, and how much of this risk is reduced as the demand variability decreases.

Here, we develop a model of the dynamics of plant production in a nursery, linked to a model for a variable demand of planting material that is satisfied by sales from nurseries and imports. We combine this model with an economic model to investigate how demand variability affects the probability of introducing new tree pathogens.

## 2 MATERIALS AND METHODS

Tree production in a nursery goes through several growth stages before plants reach a saleable size. We link this production process to economic variables such as tree maintenance cost and import costs, selling prices, and demand variability. With this information, we investigate how the probability of introducing an invasive pathogen is linked to (1) the market demand, (2) production cost and import costs, and (3) nursery owner decisions on tree production volume.

The details and mathematical formulation of the described model are found in Supporting Information Appendix S1. Here, we summarize the components and the dynamics of the model.

### 2.1 Tree population growth model in the nursery

**Figure 1:**
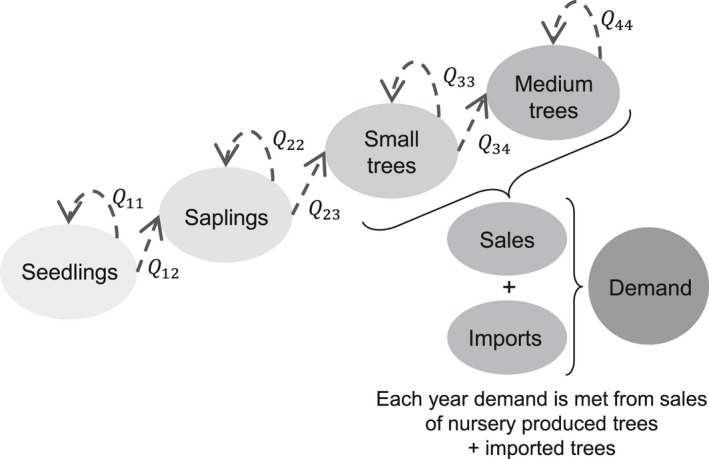
Trees are planted and grow through various stages of survival, transition *Q*_*ij*_ and no‐transition *Q*_*ii*_ rates. Once they reach a size fixed by the market demand, they are sold. If the demand is higher than the production in any given year, trees are imported. If the production is larger than the demand, the unsold stock is kept for the next selling cycle or discarded in accordance with the nursery business practice

The dynamics of the tree population in the nursery are described in time steps of 1 year. Each year, *B* seedlings or cuttings are planted. Seedlings develop into saplings, then grow to become small trees and subsequently develop into medium size trees (Figure 1). Trees in each growth stage may remain in that stage for more than 1 year. The dynamics of this tree population is described by a Lefkovitch matrix model (detailed in Supporting Information Appendix S1) where trees can grow to the next size class or remain in the same size class (*Q*_*ij*_ values in Figure 1).

### 2.2 Tree sales and imports

Trees are sold from the small and medium tree sizes shown in Figure 1. If in a year the number of trees in these size classes is smaller than the demand, trees are imported to match the difference between tree stock and demand. If after sales the number of small or medium‐sized trees is larger than the demand, the grower is left with a stock of unsold trees. These unsold trees are either left in the stock for another year, discarded, or a combination of these two. In the main text, we only analyse the case where unsold trees are discarded. In the Supporting Information Appendix S2, we analyse the case where unsold trees are maintained in stock, and show that the qualitative conclusions from our study remain unchanged, though some quantitative differences arise.

### 2.3 Demand

The demand for trees is a random variable. Each year, the demanded number of trees is drawn from a homogeneous distribution with mean demand *μ* and a standard deviation of (2*α)*/√12. Due to the absence of detailed data on the demand distribution, we use a uniform distribution. Moreover, the example analysed in the manuscript is drawn from a homogeneous distribution. However, in earlier simulations, we investigated the effect of a normal distribution on the results and found that there is no qualitative difference. This can be seen in Supporting Information Appendix S4.

### 2.4 Import of exotic diseases

The probability of importing an invasive pathogen is linked to the number of imports acquired over time. Each imported tree has a very small probability of being infected with an exotic pathogen that goes unnoticed during the import process. We calculate the mean probability per year that a tree infected with the pathogen is imported. The rationale is that when infected trees are imported more frequently the probability that this disease escapes and causes an epidemic also increases. Our models also generalize to the case of imported pests in which there is a low probability of detection on imported trees.

### 2.5 The cost calculations

Nursery growers aim to maximize their profit. Every year, the nursery owners must decide what their production volume will be. This production volume depends on production costs and on the expected demand. We take the number of yearly planted seeds or cuttings, *R*, as the main decision point for growers to maximize their profit.

The costs that a nursery owner incur are the cost of seedling planting material, the costs of tree maintenance (which can vary with size class), and the cost of importing a tree when demand is larger than stock.

**Figure 2:**
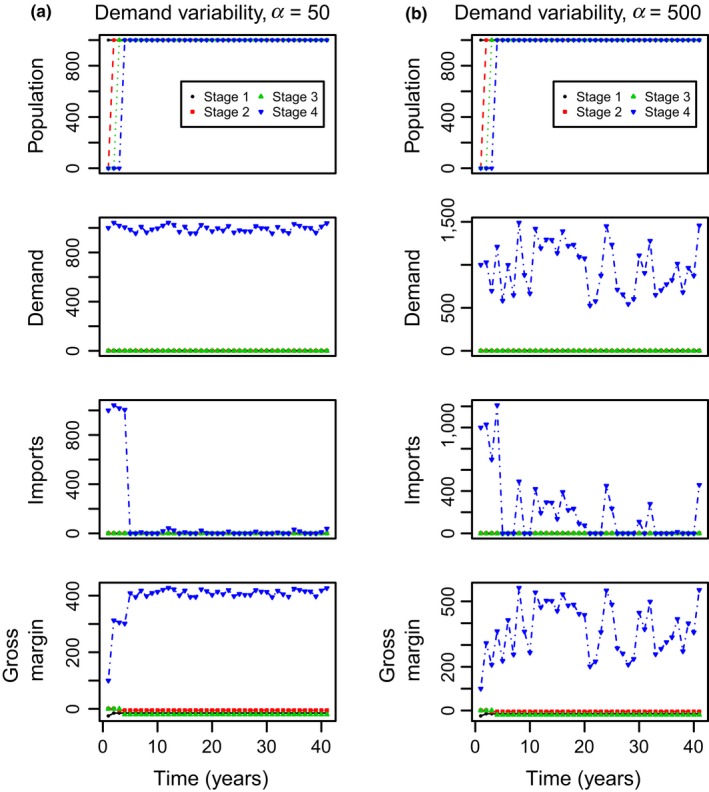
System dynamics over time for four population stage classes going from seeds (dots) to medium trees (inverted triangles) with a 100% trees survival rate. The planting rate is *R *= 1,000, the demand variability is *α = *50 and 500 for (a) and (b) respectively, and the mean demand in both cases is *μ*_4_ *= *1000 trees per year. In this system, the production and importing costs of one tree are 0.075 units per tree and 0.15 units per tree, respectively. The selling price is 0.25 units per tree. After sales, all remaining trees are discarded

**Figure 3:**
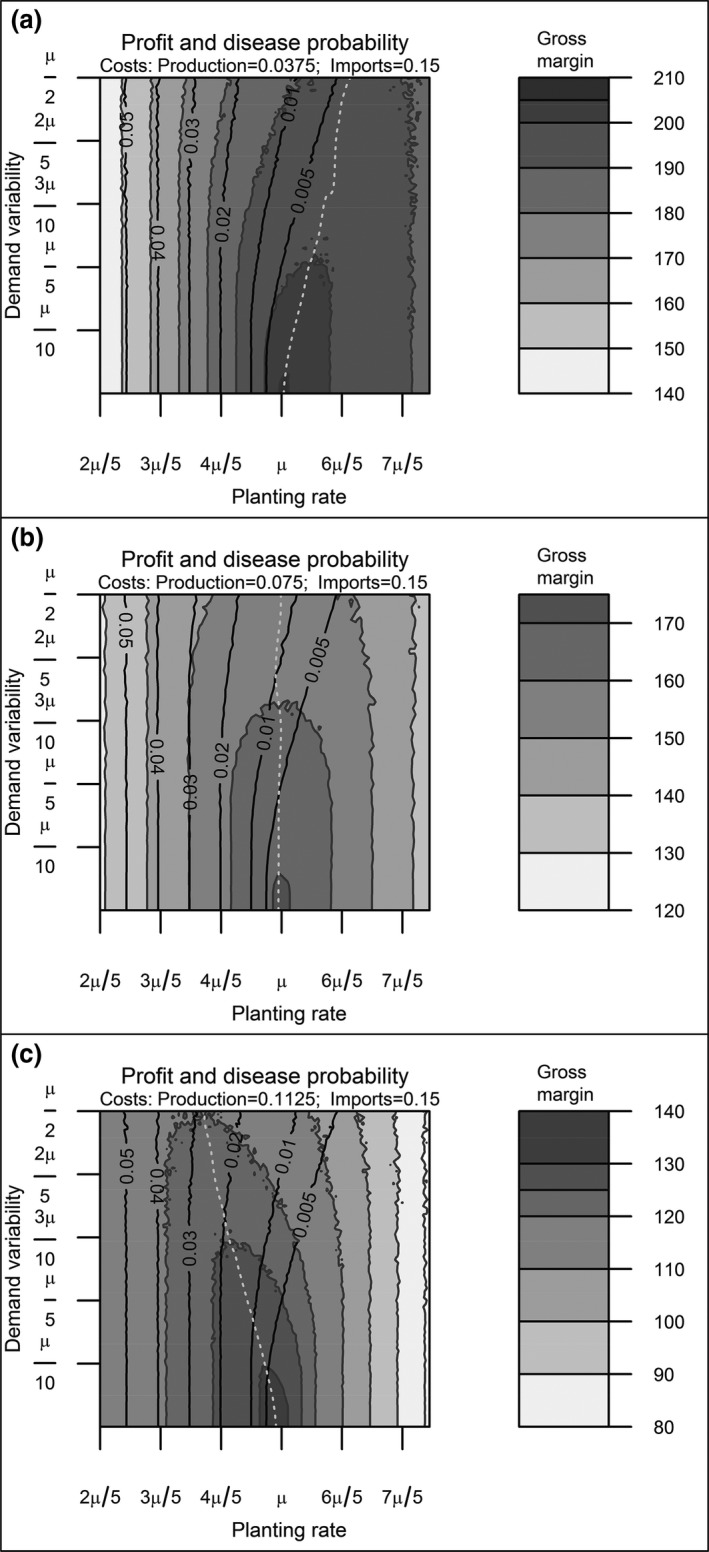
The contours shown are the result of the dynamics of a nursery with a population moving through four stages with 100% tree survival and transition rates. Sales take place only for trees in growth stage four and no trees are kept after sales. In this system, the expected average tree demand is *μ*_4_ *= *1,000 trees per cycle, the demand variability ranges between *α *= [0,500] trees per cycle, equivalent to *α = *[0, *μ*/2] in terms of the mean demand and the planting rate varies between *R *= [400, 1,500] trees per cycle, equivalent to *R* = [2*μ*/5*μ*] in terms of the mean demand. Import costs amount to 0.15 units per tree and base production costs are 0.0375 (a), 0.075 (b), and 0.1125 units per tree (c). Shaded regions show gross margin contours with respect to demand variability and planting rate. Black lines display contours of the probability of introducing an exotic disease. The grey dotted line shows where the maximum gross margin is obtained

Nursery owners obtain some profit from selling both the home‐produced and the imported trees. We refer to the captions of Figures 2 and 3 for the costs used in the general analysis and to Table 1 for the costs used in the case study. Although the cost values used in Figures 2 and 3 are arbitrary, these are in the range of values that are reasonable for this system and are based on personal communication with growers. These figures show the dynamics of hypothetical systems. The cost values used in the study case are obtained from nursery growers and the literature.

**Table 1:** Parameter values for tree growth and economic variables for Common Oak (*Quercus robur*) in the UK. Within a nursery, Common Oak survival in nurseries where most external variables are controlled, mean survival of planted acorns reaches about 90% (Kormanik, Sung, Kormanik, Schlarbaum, & Zarnoch, 1998; Moustakas & Evans, 2015). Seedlings reach between 30 and 40 cm in average by the end of the growing season (Kühne & Bartsch, 2007; Mariotti, Maltoni, Jacobs, & Tani, 2015; Turcsán et al., 2016; Valkonen, 2008; Welander & Ottosson, 1998). We then assume that tree survival reaches 90%. From this 90% survival, we also assume that 80% of the seedlings reach up to 40 cm and 20% reach heights over 40 cm

Parameter	Description	Default parameter values
		Trees sizes: 0–20 cm, 20–40 cm and 40–60 cm
*R*	Planting rate per year (thousand trees)	*R* = 400–6,000
*T*_*ij*_	Transition rates per year	*T*_12_ = 1.0 *T*_23_ = 0.8 *T*_34_ = 0.2
*S*_*ii*_	Rates of no transition per year	*S*_11_ = *S*_22_ = *S*_33_ = 0
*S*_*44*_	Holding rates per year	*S*_44_ = 0.0
*A*_*i*_	Survival rates per year	*A*_1_ = 0.90 *A*_2_ = *A*_3_ = *A*_4_ = 1
*μ*_*i*_	Mean demand per year (thousand trees)	*μ*_3_ = 2,861 *μ*_4_ = 715
*α*_*i*_	Demand variability per year (thousand trees)	*α*_3_ = 0–2,000 *α*_4_ = 0–500
*σ*	Seed costs per tree (£)	σ = 0.035
ν_*i*_	Production costs per tree (£)	ν_3_ = 0.07 ν_4_ = 0.0
*γ*_*i*_	Importing costs per tree (£)	*γ*_3_ = 0.15 *γ*_4_ = 0.20
*G*_*i*_	Selling price per tree (£)	*G*_3_ = 0.40 *G*_4_ = 0.45

Economic variables (seed, production, importing, and selling costs) and holding rates were obtained from a combination of (a) published data, (b) statistics on the web, and (c) information provided by nursery growers with Common Oak production for the forestry business. The economic variables specified in this section consider maximum costs and prices obtained from different nurseries, so this may vary slightly from nursery to nursery. Seed planting costs approximately 3.5 p per tree and overall tree production costs approximately 10.5 p per tree. There is no cost associated with production between stages 3 and 4 as trees will either reach one height or another in the same period. Import costs are approximately 15 p per tree (stage 3) and 20 p per tree (stage 4) and selling prices are approximately 40 p per tree (stage 3) and 45 p per tree (stage 4). Planting rate and demand variability are taken from data on the number of trees planted from 1976 to 2017 from the Forestry Commission statistics. Mean demand is calculated from data of the last 30 years to 2017, that is, from 1987 to 2017, where demand is substantial. Planting rate numbers are taken from the full range to show a wider range of solutions.

As we are not including overheads and operating expenses in our calculations, we calculate the gross margin instead of the profit of the nursery. The gross margin or gross profit of a nursery is calculated as the difference between income from the sales and the costs of producing and importing trees. We express the gross margin as the yearly mean gross margin of a nursery and we will assume that the nursery owner aims to maximize the expected (mean) gross margin.

## 3 RESULTS

We quantify how the probability of importing an exotic disease is affected by the planting rate and the demand variability. To this end, we first simulate the dynamics of the growth of a tree population in a nursery over time. We also simulate how demand variability and imports affect the economic dynamics of the nursery. Combining these two, we obtain contour plots of the mean gross margin as a function of the demand variability and the planting rate chosen by the nursery owner. Linking these results with a contour plot of the mean probability to import an exotic pathogen, we see how demand variability and the aim of the grower to maximize the nursery gross margin affect the probability to introduce an exotic disease.

### 3.1 The dynamics of the system

Figure 2 shows the dynamics of the system when variability in demand is comparatively small (Figure 2a) or large (Figure 2b) for the same average demand. In this simulation, medium‐sized trees are sold and surplus trees are discarded at the end of the season. In the Supporting Information Appendix S2, we show simulations where both small and medium‐sized trees are sold and where surplus trees are kept in the nursery. In Supporting Information Appendix S2 we show that when the demand variability is small, there are small variations in gross margin, demand, and imports over time. In this case, there is a slight increase in the production of trees over time due to the holding strategy adopted. When the demand variability is large, the production of trees increases over time. In turn, the number of imports over time is reduced but the costs associated to maintain trees for longer periods lead to a decrease of the gross margin over time.

Figure 2 shows time series of population, imports, costs, and demand for a system where all unsold trees are disposed of. The figure shows a system with a transient period of establishment during which trees are growing. After this period, the business is fully established and a constant influx of fully grown trees exists. All the calculations for the contour plots shown in this study are performed over the established phase where the extra costs of the nursery start‐up are not considered. When the demand variability is small, with a coefficient of variation of demand of 5% (Figure 2a) there is a small variation in gross margin, imports, and demand. In this case, the mean demand adds up to a number close to the number of trees planted, therefore, there are few imports. When the variability in demand is substantially large, that is, when this variability has a coefficient of variation of 50% (Figure 2b) the number of imports increases due to the sudden increase in tree demand at random times. Mean gross profit slightly decreases due to an increase in the number of imported trees as well as the number of discarded trees which constitute a loss.

### 3.2 Probability of introducing a disease as affected by demand and planting rate

Figure 3 shows how the mean nursery gross margin and the probability of introducing an exotic pathogen are affected by demand variability (*α*) and planting rate (*R*). The dotted white line represents the maximum gross margin obtainable.

Considering first the gross margin contours, we see that for each level of demand variability there is a planting rate that maximizes the gross margin. For the parameter values with small demand variability, this planting rate is the same as the demand rate because in these simulations trees are assumed not to die. We have run simulations with other parameters values and concluded that our qualitative conclusions do not change. For any fixed planting rate, the gross margin always decreases as the demand variability increases. Interestingly, the planting rate that maximizes the gross margin can either decrease with increasing demand variability (Figure 3a) or increase with increasing demand variability (Figure 3c). From the range of simulations developed, we conclude that the key determinant of whether at a maximum gross margin the planting rate increases or decreases with demand variability depends on the balance between the production cost of a tree in a nursery and the cost of importing a tree. For the parameters used in these simulations, we show in Figure 3 that as demand variability increases: (a) when production costs are less than half the importing costs (*κ*_*P*_ *< *(*κ*_*I*_/2)), optimum gross margin contours bend towards planting rates larger than the mean demand, (b) when production costs are larger than half the importing costs (κ_*P*_ *> *(*κ*_*I*_/2)), optimum gross margin contours bend towards planting rates smaller than the mean demand, and (c) when production costs are equal to half the importing costs (*κ*_*P*_ *= *(*κ*_*I*_ */*2)), the gross margin contours do not bend from the mean demand planning rate. In Supporting Information Appendix S3, we find that when the production costs are half the price or less than the importing costs for the parameters used in this study, producing trees with no imports is cheaper than producing and importing trees. This agrees with the results shown in Figure 3, where our gross margin is also proportional to the ratio between production and importing costs. Exactly where the balance between production and import costs switches such that the optimal gross margin line in Figure 3 changes from bending forward to bending backwards with respect to the nursery planting rate is a matter of parameter values and needs to be assessed on a case‐by‐case basis.

Now let us consider the contours of yearly mean probability to import an infected tree (black lines in Figure 3). These contours are independent of production costs and other parameter values (they are solely related to the number of imports), so the probability of importing an infected tree decreases with increasing planting rate if any proportion of the planting is ever imported. Equally, increasing the demand variability increases the probability of importing an infected tree. However, as discussed earlier, the nursery owner will aim to maximize the nursery gross margin. Considering this, combining the results for the optimal gross margin line and the contours of the probability of importing an infected tree show that there are three distinct cases:

1. If the cost of producing a tree in a nursery is less than half the cost of importing a tree (Figure 3a), the demand variability has little effect on the mean yearly probability of importing an infected tree. In this case, the contours of importing an infected tree follow the same shape as that of the optimal combination of planting rate and demand variability to optimize the gross margin.
2. If the cost of producing a tree in a nursery is equal to half the cost of importing a tree (Figure 3b), we see that the optimum gross margin is independent of the demand variability. Nonetheless, the mean yearly probability to import an infected tree increases as this probability increases with demand variability.
3. When the cost of producing a tree in a nursery is larger than half the cost of importing a tree (Figure 3c), the situation is very different. Following the optimal gross margin line, we see that an increased demand variability drastically increases the mean yearly probability to import an infected tree.

These results show that the nursery tree production costs relative to import costs determine whether an increased variability in demand increases the risk of importing an exotic disease. In the following, we explore the results for an example of a specific case of high practical relevance in the UK.

### 3.3 Case study for common oak (*Quercus robur*)

In this section, we use English or pedunculate oak (*Quercus robur*) as a study case due to its’ ecological and economic importance for the sustainability of English broadleaved forests. Broadleaves account for about half (49%) of the UK woodland area (Forestry Commission, 2011; Forestry Commission GB, 2017). Among broadleaf woodlands, the most common species found in woodland are oak (sessile and pedunculate), beech, ash, sycamore, and birch (Forestry Commission, 2012).

Oak forests provide a habitat rich in biodiversity, supporting more life forms than any other native tree. For example, their acorns are a direct food source for deer and badgers, among other species. They also host hundreds of insect species, thus providing food for birds, bats, etc. Economically speaking, oaks produce one of the hardest and most durable timbers, being the primary ship building material until the mid‐19th century. Top quality logs are used for veneer and other high‐quality timber in furniture, panelling, flooring, and ships’ planking (Kerr & Evans, 1993; (Woodland Trust, 2017).

However, oak also represents the largest number of imported species since 2013. According to UK government statutory notification data, between 2013 and 2015 there were 1,597,567 trees imported, of which 1,117,696 were oak (Quercus species) (The Horticultural Trades Association, 2017). This puts oak at risk from potentially devastating diseases, on the scale of other tree diseases such as Dutch Elm disease or Ash dieback.

The study case is built upon limited information about the growth and commercialization of Common Oak (*Q. robur*) in the UK. The nursery sector chosen is nursery growers selling and trading tree sizes between 20–40 cm and 40–60 cm which is one of the most commonly sold oak planting material in the UK. Table 1 summarizes the parameters used for this case study.

We obtained a time series of the number of broadleaf trees planted from 1976 to 2017 from the Forestry Commission statistics (Forestry Commission GB, 2017). We do not include commercial and private data in this study, as there is very little information available. There is no time series of planted oak trees available, so we take the time series of the total area of newly planted and restocked trees in the UK and estimate the number of newly planted broadleaves in the UK. From Forestry Commission (2016), we know that in the last 16 years the total coverage of oak represents between 16% and 23% of the total area covered by broadleaves. Here then, we assume that the demand for oak over time is 20% of the total of planted broadleaves during the period 1976–2017. We also assume that approximately 1,500 trees per ha are planted (Priestley & Sutherland, 2016). From these data, we obtain the average demand. We set the range of demand variability as the difference between the minimum and the maximum year‐to‐year difference in planting rates obtained. This gives the values of α_*i*_ in Table 1, where α_*i*_ is a measure of the demand variability of trees in stage *i*. The demand at any given year is a random variable taken from the uniform distribution, so that the demand is a random number drawn from the interval (*μ*_*i*_ *–α*_*i*_ *, μ*_*i*_ *+ α*_*i*_) at year *t* and *μ*_*i*_ is the mean demand of trees from stage *i*.

### 3.4 The dynamics of the system

**Figure 4:**
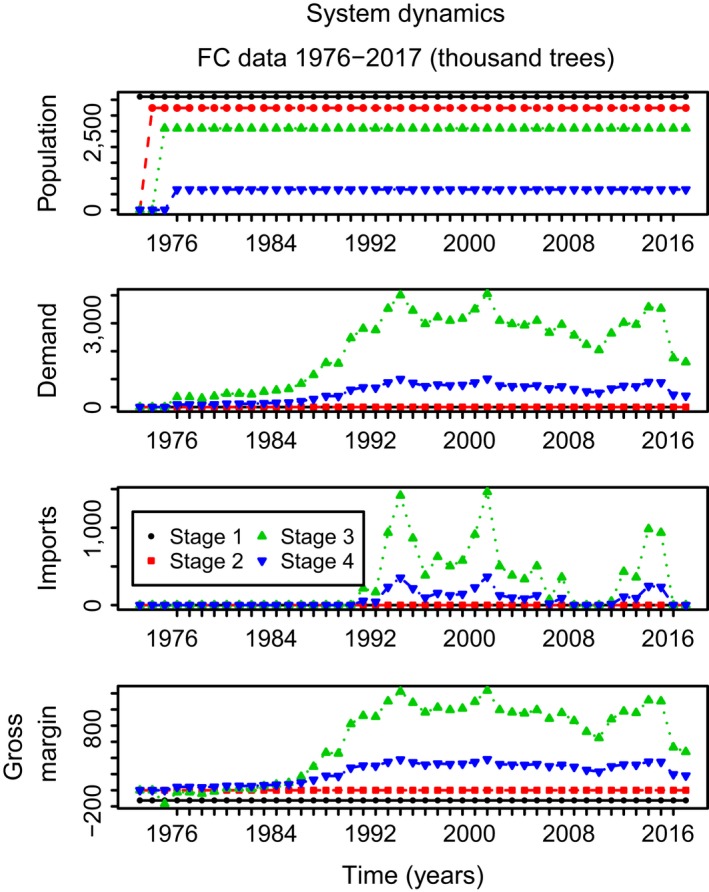
Economic variables over time for four population stage classes going from seeds (growth stage 1) to tree sizes of 20–40 (growth stage 3) and 30–50/40–60 cm (growth stage 4). Demand variability is determined by planting tree data from 1976 to 2017. After sales, remaining trees are discarded. Large variations in demand over time result in many imports throughout the last 20 years due to the sudden increase in tree demand at several points

**Figure 5:**
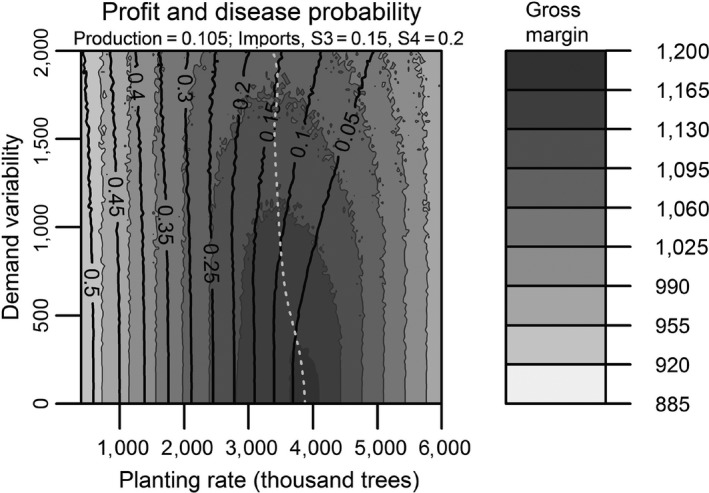
Contours of gross margin dependent on demand variability and planting rate for a study case with Common Oak as commercialized species. The production costs (£0.105 per tree) are smaller but comparable to importing costs (£0.15 and 0.20 per tree for trees in stages 3 and 4 respectively). Optimum gross margin contours (in thousand pounds units) bend towards smaller than the mean demand planting rates with increasing demand variability. The crossover between optimum gross margin line and the probability of introducing a pathogen contours is large. Invariably, as the demand variability increases, total costs increase

Figure 4 shows the dynamics of the tree population in the nursery, the demand and gross margin as well as the imports of oak from 1976 to 2017. The demand variability has led to several spikes in the number of imported trees, interspersed with a period of low import rates. Because the tree production costs are smaller than, but close to the costs of importing a tree, a large demand variability implies that there is a substantial risk of increased costs due to discarding trees not needed to cover the demand. This, in turn, implies that maximum gross margin is reached by planting fewer seeds/cuttings than the average demand when demand variability is large (Figure 5). When growers operate their nursery to maximize gross margin, this implies that an increased demand variability will sharply increase the probability of importing infected trees and hence the risk of introducing an exotic disease. For example, in this case study, we see that the probability of introducing a disease increases from 0 to 0.15 when the demand variability is increased from 0 to about half the mean demand, if we follow the line of optimum gross margin. We conclude that to protect our forests from exotic invading oak pathogens, the variability in demand for oak planting material should be reduced as much as feasible.

## 4 DISCUSSION

To prevent exotic forest pathogens from entering a country a range of tools have been developed and applied. Pathway models (Douma et al., 2016 and references therein) are used in pest risk assessment enabling monitoring and quarantine actions (Burgess & Wingfield, 2002; Luvisi, Nicolì, & Bellis, 2017; Rimbaud et al., 2015) to target the highest risk pathways of entry. Such methods do not guarantee that no infected trees are imported and enter the trade‐network. Phytosanitary inspections in trade networks and local eradication of infected stock then can prevent the pathogen from escaping into forests (Eschen et al., 2015; Koch, Yemshanov, Haack, & Magarey, 2014; Shaw & Pautasso, 2014). When a pathogen does escape from a trade‐network, the final defence is to organize an eradication campaign within infected forests.

Tree imports are an important method for tree nursery owners to cope with fluctuations in demand for tree planting material. Nursery owners plant a number seeds or cuttings each year expecting to maximize the gross margin of their nursery. The demand is however very variable (Whittet et al., 2016). Since discarding unsold trees or maintaining them in the nursery is costly, the owner tends to plant fewer than are needed to cover the demand in high demand years. To meet the demand, trees are imported which increases the risk of introduction of an exotic disease.

Risk aversion is an important factor that growers may consider to avoid bankruptcy. However, this is very sensitive to the demand distribution, which we know very little of; therefore, we do not consider it in this study. The model can explore this additional feature if the demand distribution is known. Our model quantifies the effect of demand variability on the risk of introducing an exotic disease. We have found that:

1. When a grower plants a fixed number of seeds/cuttings to cover average demand each year, an increase in the demand variability will always increase the probability of introducing an exotic pathogen.
2. When a grower adjusts the number of seeds/cuttings planted each year such that their gross margin is maximized, there are two cases to consider:
  a When the costs of producing a tree in the nursery are considerably smaller than the costs of importing a tree, the grower will adjust to higher seeding/cutting plating rates for higher demand variability. This is because discarding an unsold tree is a relatively small cost compared with the gain of selling the tree. The consequence of this is that the risk of introducing an exotic disease is hardly affected by an increase in demand variability.
  b When the cost of producing a tree in the nursery is smaller than but not very different from the cost of importing a tree, the grower will reduce the number of seeds/cutting planted each year as the demand variability increases. This is because the cost of producing and disposing of unsold trees is significant when compared to the cost of importing them. The consequence of this is that the risk of importing an exotic disease increases with increasing demand variability.

The case example of Oak production in the UK shows that in this case, an increase in demand variability from 0 to about half the mean demand results in an increase in the probability of introducing an exotic disease to about 0.15 (starting at probability 0 when demand variability is 0), if we follow the line of optimum gross margin.

There is a range of measures that can be taken by policymakers and stakeholders to manage the fluctuations in demand and the difference in cost of production and cost of import. For example, longer term grant schemes that allow nursery grower to plan their tree planting can reduce this variability. Policies that incentivize growers to sell home‐grown plants can also help to manage these fluctuations.

Our results suggest that a balanced management of demand variability and costs can significantly reduce the risk of importing an exotic forest disease.

## AUTHORS’ CONTRIBUTIONS

V.A.C., C.A.G., and F.B. conceived the project; F.B. and V.A.C. developed the model and conducted the analysis; V.A.C. led the writing of the manuscript. All authors contributed critically to the drafts and gave final approval for publication.

## DATA ACCESSIBILITY

Data are available via the Dryad Digital Repository https://doi.org/10.5061/dryad.g446vj1 (Alonso Chavez, Gilligan, & van den Bosch, 2018).
