# Supplementary figures and images for: Variability in commercial demand for tree saplings affects the probability of introducing exotic forest diseases
Source: J Appl Ecol. 2018 Aug 14;56(1):180–9. doi: 10.1111/1365-2664.13242 (PMC6334522; doi:10.1111/1365-2664.13242)

A

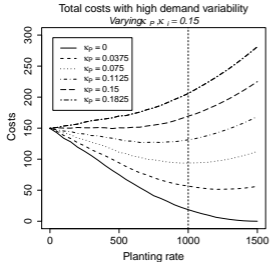

B

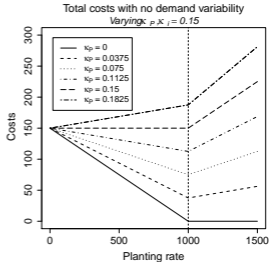

Supplement: Supplementary file 1 [file JPE-56-180-s001.pdf]

**A** Demand variability  
 $\alpha_3=50, \alpha_4=25$

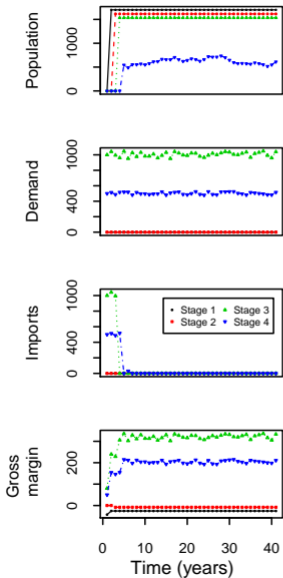

**B** Demand variability  
 $\alpha_3=500, \alpha_4=250$

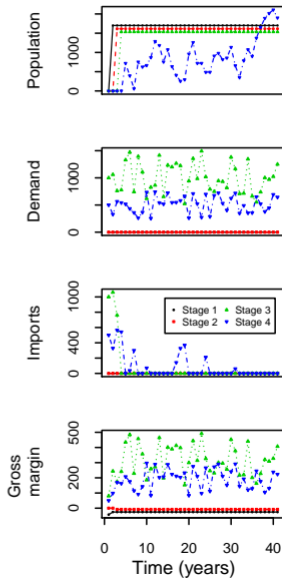

Supplement: Supplementary file 2 [file JPE-56-180-s002.pdf]

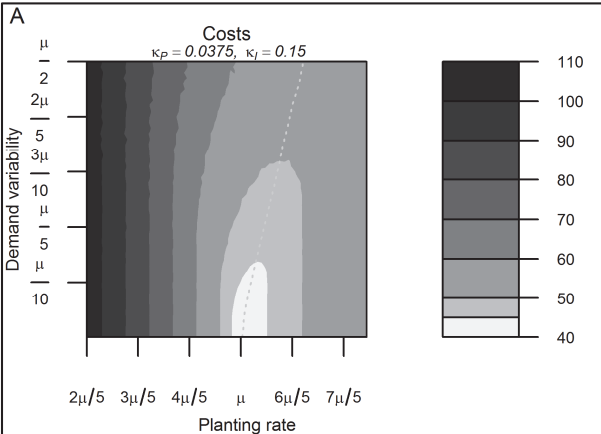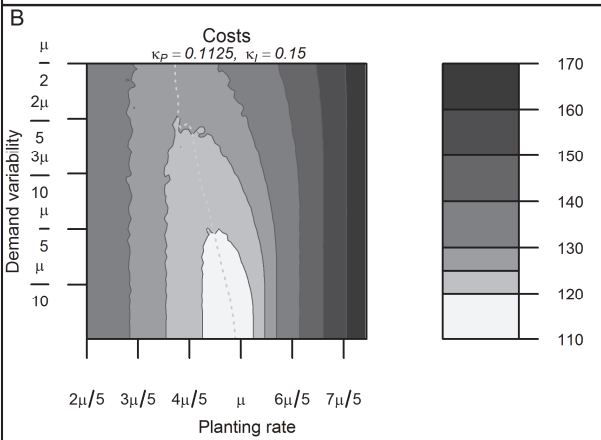

Supplement: Supplementary file 3 [file JPE-56-180-s003.pdf]
