# Supplementary material for: Variability in commercial demand for tree saplings affects the probability of introducing exotic forest diseases
Source: J Appl Ecol. 2018 Aug 14;56(1):180–9. doi: 10.1111/1365-2664.13242 (PMC6334522; doi:10.1111/1365-2664.13242)
Supplement: Supplementary file 5 [file JPE-56-180-s005.docx]

# **Appendix S1: Model description**

Consider that a population of trees is grown, sold and managed. Seeds and/or cuttings are planted every year and trees are ready to be sold once they reach a certain size. Tree demand varies from year to year so depending on the number of trees produced by the nursery in any given year, trees may need to be imported to satisfy the demand in that year. On the other hand, if the production is larger than demand, unsold trees can either be kept for future sales or discarded depending on the nursery business strategy (main text, Figure 1).

1. *Production process*

To simulate tree dynamics, we assume that the population of trees is divided into four groups consistent with their growth stage and size. The number of trees at time $t$can be written as

| $P_{t}=\left( \begin{matrix} P_{1,t} \\ P_{2,t} \\ P_{3,t} \\ P_{4,t} \end{matrix} \right)$ | (A1.1) |
| --- | --- |

where *P_1,t_*, *P_2,t_*, *P_3,t_* and *P_4,t_* are the number of seeds/cuttings, saplings, small and medium-sized trees in the nursery in year *t*, respectively. The population development is modelled using a Lefkovitch population matrix (Lefkovitch, 1965). The stage matrix is a square matrix with stage-specific rates of transition. The elements of our matrix correspond to the probabilities of transition from one stage to the next *Q_ij_* and the probabilities of not changing stage *Q_ii_*.
The Lefkovitch matrix governing the population dynamics is

| $\mathbb{L}=\left( \mathbb{T+S} \right)\mathbb{\cdot A=}$  $\left( \begin{matrix} S_{11} & 0 & 0 & 0 \\ T_{12} & S_{22} & 0 & 0 \\ 0 & T_{23} & S_{33} & 0 \\ 0 & 0 & T_{34} & S_{44} \end{matrix} \right)\left( \begin{matrix} A_{1} & 0 & 0 & 0 \\ 0 & A_{2} & 0 & 0 \\ 0 & 0 & A_{3} & 0 \\ 0 & 0 & 0 & A_{4} \end{matrix} \right)$  $=\left( \begin{matrix} Q_{11} & 0 & 0 & 0 \\ Q_{12} & Q_{22} & 0 & 0 \\ 0 & Q_{23} & Q_{33} & 0 \\ 0 & 0 & Q_{34} & Q_{44} \end{matrix} \right),$ | (A1.2) |
| --- | --- |

with 𝕋 the transition matrix where *0 < T_ij_ ≤ 1* is the probability of individuals in stage class *i* at time *t* that move to the next stage *j* at *t+1*, and *0 < S_ii_ < 1,*  the probability of individuals in stage class *i* at time *t* staying in the same stage at *t+1*. Note that *0 < S_ii_ + T_ij_ < 1* for *j=i+1,* where *j ≤ dim(𝕃-1)*. *𝔸* is the survival matrix where *A_i_* is the probability of survival of individuals in stage *i* from time *t* to *t+1*.

1. *Production dynamics with sales*

At any given year, we assume that there are sales of trees in stages 3 and/or 4. The nursery owner performs the following sequence of tasks between *t* and *t+1*.


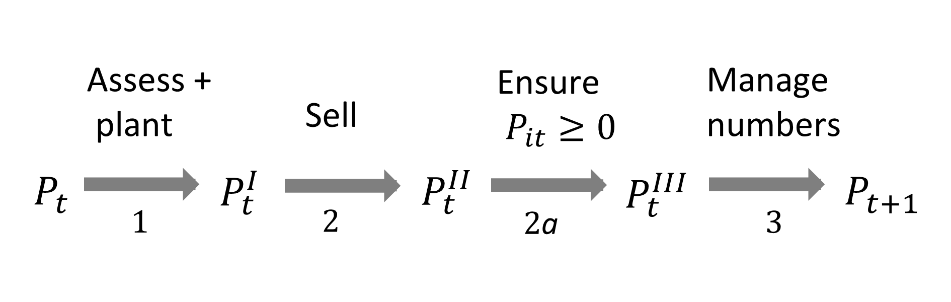


Population dynamics with sales. The superscripts indicate the action taken by the grower in the growth-sale cycle going from *t → t+1*.

1. Assess and plant trees: Seeds or cuttings are planted and these make up the *P_1,t+1_* number of planted trees. Trees that have grown are transferred to the next stage/size class. Trees that have not grown sufficiently remain in the size class they were in.
2. The grower sells trees from stages 3 and 4 matching the tree demand.

2a. If the number of trees in each class is smaller than the demand, the grower imports trees ensuring that the number of trees in the nursery is always larger or equal to zero.

1. The remaining trees are managed after the sale. There are two options here: trees are discarded or maintained in the nursery; unsold trees can be maintained in the nursery when the number of trees in stages 3 and/or 4 are larger than the demand, otherwise, these are discarded if the stock becomes too large. This is done by reducing numbers from *P_3,t_ → N_3_* and/or from *P_4,t_ →N_4_*

These nursery management steps are modelled as follows: At time $t$ the grower assesses and plant trees:

| $P_{t}^{I}\mathbb{=L}P_{t}+\bar{R}$ | (A1.3) |
| --- | --- |

where $\bar{R}$ *= (R,0,0,0)^T^* is the number of seeds/cuttings planted, 𝕃 is the Lefkovitch matrix given by Eqn. (A1.2), and superscripts indicate action taken by the grower in a selling cycle.

2. The grower sells trees in accordance with the demand at, $\bar{\xi}_{t}$ = *(0,0,ξ_3,t_,ξ_4,t_)^T^*, where *ξ_3,t_* and *ξ_4,t_* are the demand of trees in growth stages 3 and 4, respectively at time *t*. Thus,

| $P_{t}^{II}=P_{t}^{I}-\bar{\xi}_{t}$ | (A1.4) |
| --- | --- |

where *P_t_^II^* is the number of trees left after a sale takes place.

2a. Since $\bar{\xi}_{t}$ may be larger than *P_t_^I^* we shall ensure that the number of trees in the nursery is always larger or equal than zero since there cannot be a negative number of trees. Thus,

| $P_{1,t}^{III}=P_{1,t}^{II}$  $P_{2,t}^{III}=P_{2,t}^{II}$  $P_{3,t}^{III}=\left\{ \begin{aligned} P_{3,t}^{II} if P_{3,t}^{II}\geq0 \\ {0 if P}_{3,t}^{II}<0 \end{aligned} \right.$  $P_{4,t}^{III}=\left\{ \begin{aligned} P_{4,t}^{II} if P_{4,t}^{II}\geq0 \\ {0 if P}_{4,t}^{II}\leq0 \end{aligned} \right.$ | (A1.5) |
| --- | --- |

where *P_i,t_^III^* is the number of trees left after a sale considering that the number of trees in the nursery is always equal to or larger than zero. The number of trees needed to satisfy the demand when $\bar{\xi}_{t}$ > *P_t_^I^*will be completed through imports.

3. Once the grower knows the number of trees left after the sale, this number is managed. To keep a reasonable number of trees in each class, the grower either caps the number of trees that each one of the demand stages can contain or effectively discards them. Thus, the number of trees kept after a sale is given by *N_i_*, where *N_i_ ≥ 0* is a number fixed by the grower.

| $P_{1,t+1}=P_{1,t}^{III}$  $P_{2,t+1}=P_{2,t}^{III}$  $P_{3,t+1}=\left\{ \begin{aligned} P_{3,t}^{III} if P_{3,t}^{III}\leq N_{3} \\ {N_{3} if P}_{3,t}^{III}>N_{3} \end{aligned} \right.$  $P_{4,t+1}=\left\{ \begin{aligned} P_{4,t}^{III} if P_{4,t}^{III}\leq N_{4} \\ {N_{4} if P}_{4,t}^{III}>N_{4} \end{aligned} \right.$ | (A1.6) |
| --- | --- |

1. *Demand*

Variability in demand causes the number of trees needed to vary from year to year. Therefore, we define the demand as a random variable taken from a uniform distribution so that *ξ_i,t_* is a random number drawn from the interval *(μ_i_-α, μ_i_+α),* where *μ_i_* is the mean demand of stage *i* and *α* is a measure of demand variability. The demand is exactly its mean *ξ_i,t_ = μ_i_* when *α* is zero, *i.e.* when there is no variability in the demand. We also assume that max(*α*)=*μ_i_* to ensure that *α* does not take negative values.

1. *Imports*

If the demand in year *t* is larger than the number of available trees, the grower imports the difference between the demand and the number of trees available from the nursery. The total number of trees of size class 3 and 4 imported in year *t,* *I_3,t_* and *I_4,t_* respectively, are

| $I_{3,t}=\left\{ \begin{aligned} 0, if \xi_{3,t}\leq P_{3,t}^{I} \\ \xi_{3,t}-P_{3,t}^{I} , if \xi_{3,t}>P_{3,t}^{I} \end{aligned} \right.$  $I_{4,t}=\left\{ \begin{aligned} 0, if \xi_{4,t}\leq P_{4,t}^{I} \\ \xi_{4,t}-P_{4,t}^{I} , if \xi_{4,t}>P_{4,t}^{I} \end{aligned} \right.$ | (A1.7) |
| --- | --- |

Thus, the number of imports in any given year is

| $I_{t}=I_{3,t}+I_{4,t}$ | (A1.8) |
| --- | --- |

and the mean import *Î* over time is given by

| $\hat{I}=\lim_{T\to\infty} \frac{1}{T}\sum_{t=1}^{T} (I_{3,t}+I_{4,t}).$ | (A1.9) |
| --- | --- |
|  |  |

1. *Costs*

The costs associated with the sale of trees at time *t* depend on the cost of sowing a seed or cutting *(σ),* the costs of maintaining a tree of size class *i, (ν_i_)* for a year, and the cost of importing a tree of size class *i*, *γ_i_*, so:

| $\kappa_{t}=\sigma R+{{(\nu}_{1}P_{1,t}+\nu}_{2}P_{2,t}+\nu_{3}P_{3,t}+\nu_{4}P_{4,t})+(\gamma_{3}I_{3,t}+\gamma_{4}I_{4,t} )=\sigma R+\kappa_{P}+\kappa_{I}$ | (A1.10) |
| --- | --- |

where *κ_P_* are total production costs and *κ_I_* the total import costs at time *t.*

Note that we assume that base production and import costs remain fixed over time. Although this is a bold assumption, our main objective is to determine how demand variability impacts the probability of introducing invasive plant pathogens and how profit is affected by demand variability.

1. *Sales and profit*

The gain obtained by selling the trees (produced or imported) at a price *G_i_* in class *i* at time *t* is proportional to the demand in stage classes 3 and 4, so

| $G_{t}=G_{3} \xi_{3,t}+G_{4}\xi_{4,t}$ | (A1.11) |
| --- | --- |

The profit for the nursery owner is equal to the gain minus the total costs presented in equations (A1.10) and (A1.11),

| $W_{t}={G_{t}-\kappa}_{t}$ | (A1.12) |
| --- | --- |

so, the mean profit *Ŵ* obtained by the sale of trees over time is

| $\hat{W} = \lim_{T\to\infty} \frac{1}{T}\sum_{t=1}^{T} W_{t}.$ | (A1.13) |
| --- | --- |

1. *Probability of introducing invasive pathogens*

The probability of importing an invasive pathogen is linked to the number of imports acquired over time. The probability that an individual plant in an imported batch at time *t* is infected can be written as *p_ι_*. We assume that this probability is very small (*p_ι_ ≪ 1*) as plant imports are in general subject to monitoring and inspection in the importing and/or the origin country. A batch of imported plants at time *t* has *I_t_ = I_L,t_ + I_M,t_* individuals. Using the binomial distribution, we thus calculate the probability that the batch of imported trees *I_t_* at time *t* has at least one diseased individual:

| $\Pi_{t}=1-\left( 1-p_{\iota} \right)^{I_{t}}$ | (A1.14) |
| --- | --- |

The mean probability that an invasive pathogen has been introduced through imports, over time is then given by

| $\hat{\Pi}=\lim_{T\to\infty} \frac{1}{T}\sum_{t=1}^{T} \Pi_{t}.$ | (A1.15) |
| --- | --- |

Which can be approximated as

| $\hat{\Pi}=p_{\iota}\cdot\lim_{T\to\infty} \frac{1}{T}\sum_{t=1}^{T} I_{t}.$ | (A1.16) |
| --- | --- |

**References**

Lefkovitch, L. P. (1965). The study of population growth in organisms grouped by stages. *Biometrics*, *21*(1), 1. doi:10.2307/2528348
