# Supplementary material for: Variability in commercial demand for tree saplings affects the probability of introducing exotic forest diseases
Source: J Appl Ecol. 2018 Aug 14;56(1):180–9. doi: 10.1111/1365-2664.13242 (PMC6334522; doi:10.1111/1365-2664.13242)
Supplement: Supplementary file 6 [file JPE-56-180-s006.docx]

# **Appendix S2: Nursery dynamics with a tree holding strategy**

Fig. S2 shows a system with a population moving through four stages with a 95% trees survival rate. From the total number of trees in each stage, 95% of them move to the next growth stage after one year, i.e. out of 100 planted trees approximately 90 arrive at growth stage 3 and 86 to stage 4. In this case, trees not sold are kept for the next selling cycle (*S_44_* =1) and sales take place when trees are in growth stages 3 and 4.

In this example, the average tree demands are *μ_3_=1000 trees/year* and *μ_4_=500 trees/year* for trees in growth stages 3 and 4 respectively. We test how changes in the demand affect the system by adding small and large demand variabilities to the system. The corresponding demand variabilities are *α_3_ =50*, *α_4_ =25* (panel A) and *α_3_ =500*, *α_4_ =250* (panel B).

When the demand variability is small Fig. S2, panel A shows that there are small variations of the economic variables over time. The joint mean demands add up to a number close to the number of trees planted, therefore, the imports sizes over time are small. There is a slight increase in the production of trees in panel A due to the holding strategy followed by the grower.

Contrastingly, Fig. S2, panel B show that if unsold trees are kept (i.e. if *S_44_ =1*), the tree stock-rise over time increases slowly, resulting on a small number of imports over time; however, costs associated to maintain trees for longer periods result in a decrease of the profit over time.

# **Figure legends**

Figure S2. Economic variables dynamics. In this example, 1700 seeds are planted each year. The demand variabilities for trees in growth stages 3 and 4 are *α_3_ =50*, *α_4_ =25* for panel A and *α_3_ =500*, *α_4_ =250* for panel B, respectively. The mean demands for trees in stage 3 and 4 are *μ_3_ =1000* and *μ_4_ =500 trees/year* respectively. After sales, all remaining trees are carried over to the next selling cycle.
