# Supplementary material for: Variability in commercial demand for tree saplings affects the probability of introducing exotic forest diseases
Source: J Appl Ecol. 2018 Aug 14;56(1):180–9. doi: 10.1111/1365-2664.13242 (PMC6334522; doi:10.1111/1365-2664.13242)
Supplement: Supplementary file 7 [file JPE-56-180-s007.docx]

# **Appendix S3: Balance between tree production costs in the nursery and importing costs**

Assume that the total number of trees obtained from production and imports in each year is *N*, where *N* may vary from year to year. If *κ_I_ > κ_P_* then, the costs per tree decrease as planting rate *R* increases until demand *N* is reached. If the planting rate is larger than the demand *R>N*, the costs due to the production of trees increase as planting rate increases. Total costs to obtain *N* trees can then be divided as

| $\kappa_{T}N=\kappa_{P}N_{P}+\kappa_{I}N_{i} if N\leq\xi$,$\kappa_{T}N=\kappa_{P}N if N>\xi$, | (S3.1) |
| --- | --- |

where *κ_T_* represents the costs incurred to satisfy the tree demand *N*; *N_P_* and *N_I_* are the number of produced and imported trees respectively, so that, *N_P_ + N_I_ =N*. From equation (S3.1) we have that, to increase $a$ trees to the planting rate to obtain *N* trees can be written as,

| $\kappa_{T}N=\kappa_{P}{(N}_{P}+a)+\kappa_{I}{(N}_{I}-a) if N\leq\xi$,$\kappa_{T}N=\kappa_{P}{(N}_{P}+a) if N>\xi,$ | (S3.2) |
| --- | --- |

Taking derivatives with respect to the planting rate *R*, we obtain

| $\frac{d\kappa_{T}N}{dR}=\frac{\left[ \kappa_{P}{(N}_{P}+a)+\kappa_{I}{(N}_{I}-a) \right]-\left[ \kappa_{P}N_{P}+\kappa_{I}N_{I} \right]}{a}=\kappa_{P}-\kappa_{I} for N\leq\xi$ $\frac{d\kappa_{T}N}{dR}=\frac{\kappa_{P}{(N}_{P}+a)-\kappa_{P}N_{P}}{a}=\kappa_{P} for N>\xi$ | (S3.3) |
| --- | --- |

To know when producing only trees is cheaper than planting plus importing, to obtain the demanded number of trees *N*, we require that *κ_P_ ≤ –(κ_P_ – κ_I_)*, given that *κ_P_ – κ_I_ < 0*, or equivalently, that *κ_P_ ≤ (κ_I_/2)*.

Fig. S3-1 shows total costs with respect to planting rate for a system where import costs are fixed *κ_I_=0.015*, production costs are a multiple of import costs, (i.e., *κ_P_ = (0, κ_I_/4=0.0375, κ_I_/2=0.075, (3κ_I_/4)=0.1125, κ_I_=0.14, (5κ_I_/4)=0.1825))*. Panel A shows that whenever *κ_P_ < κ_I_* the minimum cost is reached when the production number is equal to the demand if demand variability is zero. Panel B shows that for larger demand variability the minimum costs are obtained when *κ_P_ ≤ (κ_I_/2)*.

*Production costs contours and demand variability*

Fig. S3-2 shows contours of total costs with respect to demand variability and planting rate for production costs *κ_P_=0.0375* (panel A) and *κ_P_=0.1125* (panel B). The cost contours represent a system with a population moving through 4 stages with a 100% trees survival and transition rates. Sales take place only for trees in growth stage 4 and no trees are kept after sales (*T_44_=1*).

In this system, the expected average tree demand is *μ_4_ =1000 trees/year*, the demand variability ranges between *α=[0,500] trees/year* and the planting rate varies between *R=[400,1500] trees/year*.

Total costs increase as demand variability increases for any fixed planting rate. On the other hand, when production costs are significantly smaller than import costs *(κ_P_ ≤ (κ_I_/2))*, contours bend towards planting rates larger than the expected mean demand as demand variability increases. Contrastingly, when production costs are comparable (even if smaller) with import costs *(κ_P_ > (κ_I_/2))*, contours bend towards planting rates smaller than the expected mean demand as demand variability increases. Light-grey dotted lines show the minimum costs with respect to planting rate.

# **Figure legends**

**Figure S3-1.** Total costs vary as production costs change and import costs remain constant. Average demand is $1000 trees/year$. Panel A shows a system without demand variability while panel B has a demand variability of $\alpha=500 trees/year.$ As planting rate increases, cost decreases until demand is met. For planting rates larger than the net demand, costs increase.

**Figure S3-2.** Contours of total costs dependent on demand variability and planting rate. When the production costs are significantly smaller than the importing costs, the minimum cost contours bend towards larger than the mean demand planting rates as demand variability increases. Conversely, when production costs are comparable to the importing costs, the minimum cost contours bend towards smaller than the mean demand planting rates as demand variability increases. Invariably, as the demand variability increases, total costs increase.
