# Supplementary material for: Variability in commercial demand for tree saplings affects the probability of introducing exotic forest diseases
Source: J Appl Ecol. 2018 Aug 14;56(1):180–9. doi: 10.1111/1365-2664.13242 (PMC6334522; doi:10.1111/1365-2664.13242)
Supplement: Supplementary file 8 [file JPE-56-180-s008.docx]

# **Appendix S4: Comparison between results using the uniform and the normal distribution to describe the demand distribution**

Here we show a comparison between two systems where the demand variability is taken from the uniform and the normal distributions, demonstrating that the results are qualitatively the same, although some quantitative differences arise. Figure S4 shows the results of a system where each year the demanded number of trees are drawn from a homogeneous distribution with mean demand $\mu$ and a standard deviation of *(2α)/√12.* (Panels A and C). Panels B and D show the results of a system where each year the demanded number of trees is drawn from a normal distribution with mean demand $\mu$ and a standard deviation of *(0.75α).*

# **Figure legends**

Figure S4. Contours of gross margin dependent on demand variability and planting rate using as a demand distribution the homogeneous (Panels A, C) and the normal (Panels B, D) distributions. The expected average tree demand is *μ = 1000 trees/cycle*, the demand variability ranges between *α = [0, μ/2] trees/cycle* and the planting rate varies between *R = [2μ/5, μ] trees/cycle*. Import costs amount to *0.15 units/tree* and base production costs are *0.0375* (Panels A and B) and *0.1125 units/tree* (Panels C and D). Shaded regions show gross margin contours with respect to demand variability and planting rate. Black lines display contours of the probability of introducing an exotic disease. The grey dotted line shows where the maximum gross margin is obtained.
