# Supplementary material for: Variability in commercial demand for tree saplings affects the probability of introducing exotic forest diseases
Source: J Appl Ecol. 2018 Aug 14;56(1):180–9. doi: 10.1111/1365-2664.13242 (PMC6334522; doi:10.1111/1365-2664.13242)
Supplement: Supplementary file 4 [file JPE-56-180-s004.pdf]

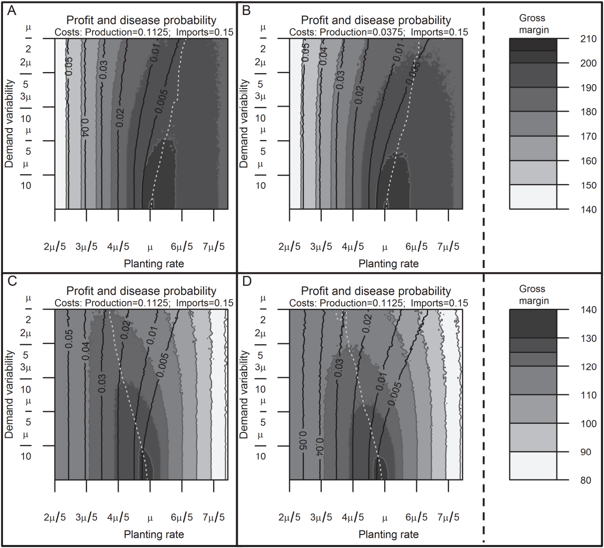
A
Profit and disease probability
Costs: Production=0.1125; Imports=0.15
B
Profit and disease probability
Costs: Production=0.0375; Imports=0.15
C
Profit and disease probability
Costs: Production=0.1125; Imports=0.15
D
Profit and disease probability
Costs: Production=0.1125; Imports=0.15
Demand variability
Planting rate
2μ/5
3μ/5
4μ/5
μ
6μ/5
7μ/5
0.05
0.04
0.03
0.02
0.01
0.005
Gross margin
210
200
190
180
170
160
150
140
130
120
110
100
90
80
